# Comparison of laparoscopic versus open simple nephrectomy in patients with xanthogranulomatous pyelonephritis: A singlecenter analysis of outcomes and predictors of surgical approaches and complications

**DOI:** 10.1097/CU9.0000000000000067

**Published:** 2022-08-02

**Authors:** Francesco Chiancone, Francesco Persico, Marco Fabiano, Clemente Meccariello, Riccardo Giannella, Maurizio Fedelini, Giovanni Lughezzani, Paolo Fedelini

**Affiliations:** a Department of Urology, A.O.R.N. A. Cardarelli, Naples, Italy; b Department of Urology, Humanitas Clinical and Research Center, IRCCS, Rozzano, Milan, Italy; c Department of Biomedical Sciences, Humanitas University, Rozzano, Milan, Italy

**Keywords:** Inflammation, Laparoscopy, Minimally invasive surgery, Nephrectomy, Stones, Xanthogranulomatous pyelonephritis

## Abstract

**Background:**

The aim of this study was to compare the outcomes of open simple nephrectomy and laparoscopic simple nephrectomy in patients with xanthogranulomatous pyelonephritis (XGP) in a single-institutional retrospective study and to identify predictive factors of surgical approaches and complications.

**Materials and methods:**

We retrospectively analyzed the data of 67 consecutive patients with a histopathological diagnosis of XGP who underwent either open simple nephrectomy (ON) or laparoscopic simple nephrectomy (LN) from January 2014 to April 2020. The primary endpoint was the evaluation of perioperative outcomes and complications. Secondary endpoints were to define factors influencing the surgical approach and the likelihood of postoperative complications.

**Results:**

Overall, 44 out of 67 patients (65.67%) underwent ON, while 23 (34.33%) underwent LN. Patients in the ON group experienced more postoperative pain according to the visual analogic scale *(p =* 0.032). Moreover, time to deambulation and time to return to full daily activities, assessed according to the 12-Item Short Form Survey physical and mental component summary scores questionnaires, were significantly shorter in the LN group *(p* = 0.021, *p <* 0.001, and *p <* 0.001, respectively). Of note, there were no significant differences in intraoperative and postoperative complication rates among the groups *(p* = 0.258 and *p* = 0.317, respectively). No conversion to open surgery was described. Logistic regression analysis demonstrated that urgency *(p* = 0.025) was the only predictor associated with a higher risk of intraoperative complications. However, no independent factors associated with postoperative complications or with the surgical approach of choice were found.

**Conclusions:**

Based on our results, laparoscopic treatment of XGP represents a feasible alternative to ON, resulting in less postoperative pain and faster recovery. In skilled hands, LN should be considered as the treatment of choice for XGP.

## 1. Introduction

Xanthogranulomatous pyelonephritis (XGP) is a rare suppurative reaction characterized by renal parenchyma destruction and its replacement by granulomatous tissue containing xanthomatous cells. Xanthogranulomatous pyelonephritis was described for the first time by Schlagenhaufer in 1916 using the term “Staphylomycosis”. In 1944, Osterlin coined the name “Xanthogranuloma”.^[1]^

Xanthogranulomatous pyelonephritis represents from 0.6% to 1% of all cases of kidney infections, and it may be either diffuse (85%) or focal (15%). Xanthogranulomatous pyelonephritis is more common in women than in men, with a peak of incidence within the fifth and sixth decades of life.^[1]^ In rare cases, younger people may be affected by XGP.^[2]^

Xanthogranulomatous pyelonephritis is usually associated with urinary obstruction, most often caused by urolithiasis, urinary tract obstruction and/or infections, diabetes, and immunodeficiency. The pathogens most frequently associated with XGP are *Enterococcus faecalis, Escherichia coli, Pseudomonas, Proteus mirabilis,* and *Klebsiella.*^[3]^

Typical presentation of XGP includes recurrent fever, asthenia or weight loss, urinary symptoms such as hematuria or stranguria, and flank pain.^[1]^ In most cases, renal function is severely impaired.

On imaging, the kidney is usually enlarged, with a thin parenchyma, with or without stones and/or an intrarenal or perirenal fluid collections. A computed tomography (CT) finding of multiple rounded hypoechoic areas around the contracted renal pelvis, described as the “bear paw sign,” is considered the main radiological characteristic associated with XGP.^[4]^ However, XGP may also mimic renal cell carcinoma.^[5]^ Similar to a malignancy, the associated inflammatory process can extend to perinephric tissues and adjacent organs (especially the duodenum) leading to obliteration of tissue planes and causing fistulisations.^[6,7]^

In addition to these radiological findings, the diagnosis of XGP depends on the histopathological analysis of the excised kidney. Although antibiotics can be useful in acute infections, open simple nephrectomy (ON) represents the optimal treatment for XGP. However, ON is associated with a higher risk of surgical site infections, increased postoperative pain, longer hospitalization, and prolonged convalescent periods.^[8]^

Moreover, the inflammatory involvement of the renal parenchyma and of the adjacent structures can make surgery extremely challenging. Initial reports in minimally invasive treatment of inflammatory kidney diseases showed high conversion and complication rates.^[9]^ While current guidelines do not define a surgical approach of choice for XGP, recently there has been a changing trend in favor of laparoscopy. Several papers demonstrated that laparoscopic simple nephrectomy (LN) can be safe and effective when carried out by skilled surgeons.^[10]^ Furthermore, LN is related with lower blood loss and shorter hospital stay when compared to ON.^[11]^

Since only a few comparative studies with a small sample size have been published, we aimed to compare the outcomes of ON and LN in XGP patients within a single-institutional retrospective study identifying predictive factors of surgical approaches and complications.

## 2. Materials and methods

For this single-center observational study, we retrospectively analysed the data of 67 consecutive subjects with a histopathological diagnosis of XGP who underwent either ON or LN between January 2014 and April 2020. Demographic and clinical variables were collected for all patients. Data concerning perioperative outcomes and complications were also available in all cases. All subjects underwent a preoperative CT scan.^[12]^ The extension of the inflammatory process to the perinephric space and to others organs was evaluated according to the Malek & Elder classification^[13]^ as follows: the inflammatory process is confined to the renal parenchyma (Stage I); the inflammatory process involves the Gerota’s layer and perinephric fat (Stage II or perinephric); and the inflammatory process extends beyond the perinephric fat involving retroperitoneal and surrounding structures (Stage III or paranephric).

Intraoperative and postoperative complications were categorized and described according to the Satava^[14]^ and Clavien- Dindo systems.^[15]^ Postoperative pain was evaluated according to the visual analogic scale at 24 hours. Health related quality of life was evaluated at 1 month follow-up, using the 12-Item Short Form Survey Physical and Mental Component Summary Scores.

For the current study, we included patients with an histopathological diagnosis of XGP with a homolateral glomerular filtration rate <15mL/min and a normally functioning contralateral kidney.^[16]^ Conversely, we excluded patients with any contralateral renal disease. Patients with a history of open or laparoscopic ipsilateral kidney surgery, other causes of inflammatory disease such as interstitial nephritis, chronic pyelonephritis, and renal tuberculosis were also excluded.

The primary endpoint was the evaluation of perioperative outcomes and complications. Secondary endpoints were to define factors influencing the surgical approach and the likelihood of postoperative complications.

### 2.1. Procedures

A single surgical team performed all procedures. All patients received general anesthesia associated with the Transversus Abdominal Plane block to provide postoperative analgesia.^[17]^ Open simple nephrectomys were performed with a retroperitoneal approach, while LNs were performed using a transperitoneal technique. The ONs were performed with a lumbotomic incision, positioning the patient in extended flank position. When needed, we opened the peritoneum to allow a sufficient exposure of the colon and allowing for its detachment. All LNs were performed with the patient in a semi lateral decubitus position. The first trocar was introduced according to Hasson’s open technique at the base of the belly-button on the pararectal line. We then usually placed two additional trocars (10mm, 5mm) above the iliac spine at the anterior axillar line and at the subcostal region at Palmer’s point. A third 5 mm trocar was sometimes needed to allow liver handling. All laparoscopic procedures were performed using a 0° 3-D camera. After mobilization of the colon, we prepared the renal pedicle, along with the identification of the ureter. The elements of the renal pedicle were isolated, ligated using Hem-o-Lok clips, and transected. In the presence of significant perirenal adhesions, a Ligasure dissector was used. The whole kidney was usually excised without opening the Gerota’s fascia. Finally, the specimen was inserted into a laparoscopic bag and extracted through a small flank incision involving the lateral port. In all cases, a drainage tube and a urethral catheter were left in place.

### 2.2. Data analysis

All data were gathered in a prospectively maintained database and were retrospectively analysed. Frequencies and proportions were reported for categorical variables, while the mean, median, and standard deviation (SD) were reported for continuous variables.

Chi-squared (*x*^2^) and Mann-Whitney tests were used to calculate the statistical significance of differences in proportions and medians, respectively.

Finally, logistic regression models were fitted to test the preoperative factors having an independent influence on the surgical approach and to evaluate independent predictors of intraoperative and postoperative complications. Statistical analyses were conducted using SPSS version 23.0 (Armonk, NY, IBM Corp.), setting the statistical significance as a *p*-value < 0.05.

## 3. Results

Overall, 44 out of 67 patients (65.67%) underwent ON, while 23 (34.33%) underwent LN. Table 1 describes the demographic and clinical characteristics of the patients. No significant differences were detected among the two groups except for the cause of XGP *(p* = 0.025). As shown in Table 2, patients in the ON group were more likely to have higher postoperative pain (determined with the visual analogic scale score) compared with the LN group (*p* = 0.032). Moreover, time to deambulation (*p* = 0.021), Physical Component Scores (*p* < 0.001) and Mental Component Scores (*p* < 0.001) were significantly lower in the LN group (Table 2). Logistic regression analysis showed that no preoperative factors had a significant impact on the choice of the surgical approach (Table 3). Detailed information about complications of the two groups and their management are reported in Table 4 and 5. Interestingly, we observed no significant differences in intraoperative (*p* = 0.258) and postoperative (*p* = 0.317) complication rates between ON and LN cases. Two severe complications occurred during the early postoperative period. In the ON arm, there was a septic embolism to the contralateral renal artery. Despite the endovascular recanalization of the renal artery, the impairment of the kidney function led the patient to hemodialysis. In the LN group, there was one case of septic shock that progressed towards exitus.

**Table 1:** Comparison of categorical and continuous variables between open and laparoscopic groups.

Categorical variables, n (%)		Open (n = 44)	Laparoscopy (n = 23)	*p*
Gender				
Male		23 (52.27%)	12 (52.17%)	0
Female		21 (47.73%)	11 (47.83%)	0.994
Diabetes				
No		29 (65.91%)	13 (56.52%)	0.451
Yes		15 (34.09%)	10 (43.48%)	0.451
Immunosuppression				
No		37 (84.09%)	22 (95.65%)	0.166
Yes		7 (15.91%)	1 (4.35%)	0.166
ASA score				
2		18 (40.9%)	7 (30.4%)	0.391
3		25 (56.8%)	14 (60.9%)	0.391
4		1 (2.3%)	3 (8.7%)	0.391
Causes			
Stones		40 (90.9%)	16 (69.6%)	**0.025**
Stenosis		4 (9.1%)	7 (30.4%)	**0.025**
M&E classification			
1		25 (56.8%)	11 (47.8%)	0.197
2		7 (15.9%)	8 (34.8%)	0.197
3		12 (27.3%)	4 (17.4%)	0.197
Side			
Right		27 (61.4%)	18 (78.3%)	0.162
Left		17 (38.6%)	5 (21.7%)	0.162
Pain			
No		37 (84.1%)	18 (78.3%)	0.555
Yes		7 (15.9%)	5 (21.7%)	0.555
Fever			
No		21 (47.7%)	14 (60.9%)	0.307
Yes		23 (52.3%)	9 (39.1%)	0.307
UTI			
No		33 (75%)	16 (69.6%)	0.634
Yes		11 (25%)	7 (30.4%)	0.634
Macroscopic hematuria			
No		42 (95.5%)	22 (95.7%)	0.970
Yes		2 (4.5%)	1 (4.3%)	0.970
Asthenia			
No		38 (86.4%)	21 (91.3%)	0.554
Yes		6 (13.6%)	2 (8.7%)	0.554
Positive urine culture			
No		37 (84.1%)	17 (73.9%)	0.317
Yes		7 (15.9%)	6 (26.1%)	0.317
Organisms found in cultures				
* E. coli*		4 (57.1%)	1 (16.7%)	0.144
* Klebsiella*		1 (14.3%)	4 (63.7%)	0.144
* Pseudomonas*		2 (28.6%)	1 (16.7%)	0.144
Preoperative drainage			
No		9 (20.5%))	7 (30.4%)	0.652
Nephrostomy tube		14 (31.8%)	6 (26.1%)	0.652
Ureteral stenting		21 (47.7%)	10 (43.5%)	0.652
Surgical planning			
Elective		39 (88.6%)	18 (78.3%)	0.258
Urgent		5 (11.4%)	5 (21.7%)	0.258
**Continuous variables**		**Group (n)**	**Mean rank**	***Z***	**Sig**
Age, yr		Open (44)	32.56	−0.839	0.401
		VLS (23)	36.76		
BMI, kg/m^2^		Open (44)	32.45	−0.907	0.365
		VLS (23)	36.96		
CRP, mg/L		Open (44)	37.07	−1.785	0.074
		VLS (23)	28.13		
Neutrophils, n°/mm^3^		Open (44)	34.45	−0.267	0.790
		VLS (23)	33.13		
Serum creatinine level, mg/dL		Open (44)	36.19	−1.293	0.196
		VLS (23)	29.80		

ASA = American Society of Anesthesiologists; BMI = body mass index; CRP = C-reactive protein; M&E = monitoring and evaluation; VLS = videolaparoscopy; UTI = urinary tract infection.

*p < 0.05.

**Table 2:** Perioperative outcomes in the open and laparoscopic groups.

Continuous variables	Group (n)	Mean rank	*Z*	Sig
Operation time, min	Open (44)	33.52	−0.278	0.781
	VLS (23)	34.91		
Blood loss, mL	Open (44)	31.43	−1.512	0.131
	VLS (23)	38.91		
Postoperative intensive care stay, d	Open (44)	31.89	−1.524	0.127
	VLS (23)	38.04		
Postoperative hospital stay, d	Open (44)	34.76	−0.453	0.651
	VLS (23)	32.54		
VAS scale PACU, score	Open (44)	33.66	−2.138	0.032
	VLS (23)	26.02		
Drains time, d	Open (44)	32.82	−0.116	0.908
	VLS (23)	33.38		
Patency of the intestinal tract, hr	Open (44)	33.90	−0.573	0.567
	VLS (23)	31.12		
Time to ambulation, d	Open (44)	36.51	−2.308	0.021
	VLS (23)	25.64		
PCS-12 one month, points	Open (44)	27.48	−3.791	<0.001
	VLS (23)	46.48		
MCS-12 one month, points	Open (44)	26.48	−4.372	<0.001
	VLS (23)	48.39		

MCS-12 = Mental Component Scores; PACU = Post Anesthesia Care Unit; PCS-12 = Physical Component Scores; VAS = Visual Analogue Scale; VLS = videolaparoscopy.

*p < 0.05.

**Table 3:** Logistic regression analysis for assessing the choice of the surgical approach (open vs. laparoscopy).

Variables	OR [Exp(B)]	95% CI	*p*
Age	1.024	0.949–1.106	0.538
Gender	1.290	0.334–4.985	0.712
BMI	1.216	0.931–1.588	0.151
ASA score			
2 (reference)	‐		
3	2.259	0.540-9.449	0.264
4	12.323	0.284–534.551	0.192
Causes			
Stones (reference)			
Stenosis	4.113	0.681–24.847	0.123
M&E classification			
1 (reference)			
2	3.702	0.789–17.373	0.097
3	1.302	0.185–9.171	0.791
Side			
Right (reference)			
Left	0.504	0.110–2.305	0.337
Fever			
No (reference)			
Yes	0.903	0.219–3.730	0.888
UTI			
No (reference)			
Yes	1.055	0.229–4.835	0.945
Positive urine culture			
No (reference)			
Yes	3.486	0.384–31.671	0.267
Preoperative drainage			
No (reference)			
Nephrostomy	0.406	0.049–3.359	0.403
Stenting	0.632	0.113–3.532	0.601
Surgical planning			
Elective (reference)			
Urgent	1.931	0.265–14.081	0.516
CRP	0.969	0.914–1.026	0.280
Neutrophils	1	0.999–1	0.437

ASA = American Society of Anesthesiologists; BMI = body mass index; CI = confidence interval; CRP = C-reactive protein; M&E = monitoring and evaluation; OR = odds ratio; UTI = urinary tract infection.

**Table 4:** Intraoperative and postoperative complications of the open and laparoscopic groups.

Intraoperative complications according the Satava classification:							
**Open**	**Grade**	**n**	**Management**	**Laparoscopy**	**Grade**	**n**	**Management**
Intraoperative blood loss	I	1	Intraoperative hemostasis	Splenic injury	I	2	Intraoperatory laparoscopic
from adrenal gland			without need of blood transfusion				repair by hemostatic agents
Diaphragmatic lesion	I	1	Intraoperative repair	Lumbar vein injury	II	1	Laparoscopic repair and intraoperative blood transfusion
Colic injury	I	2	Intraoperative repair	Duodenal injury	II	1	Intraoperative repair, parenteral nutrition and nasogastric tube for gastric decompression
Pneumothorax	II	1	Chest tub	Diaphragm injury	II	1	Intraoperative repair
Overall		5 (11.4%)				5 (21.7%) *p* = 0.258	

**Table 5:** Postoperative complications according to the Clavien-Dindo system.

**Open**	**Grade**	**n**	**Management**	**Laparoscopy**	**Grade**	**n**	**Management**
Postoperative pain	I	2	Opioid analgesics	Urinary retention	I	1	Re-catheterization
Postoperative anemia without additional complications	II	2	Blood transfusions	Postoperative anemia without additional complications	II	1	Blood transfusions
Abdominal wall infection	II	1	New generation parenteral antibiotics	Paralytic ileus	II	2	Nasogastric tube placement+ trimebutine+parenteral nutrition
Retroperitoneal abscess	IIIb	1	Re-laparotomy drainage and prolonged antibiotics	Deep venous thrombosis	II	1	Anticoagulants
Septic embolism of	IVa	1	Renal ischemia with acute	Septic shock	V	1	Intensive care unite treatment
contralateral renal artery			kidney injury (AKI) requiring dialysis				
Overall		7 (15.9%)		Overall		6 (26.1%) *p* = 0.317	

Comparing stages I and II versus stage III disease, no significant differences were observed for intraoperative and postoperative complications *(p* = 0.6547 and 0.1167, respectively). Moreover, comparing different surgical approaches only in patients with stage III XGP, no significant difference was observed for intraoperative complications (*p* = 0.7555), while a significant difference in favor of LN was observed for postoperative complications (*p* = 0.0455).

Logistic regression analysis demonstrated that urgency (*p* = 0.025) was associated with a higher risk of intraoperative complications (Table 6). However, no independent factors for postoperative complications were found (Table 7).

**Table 6:** Logistic regression analysis for assessing the likelihood of intraoperative complications.

Variables	OR [Exp(B)]	95% CI	*p*
Age	1.128	0.956–1.332	0.154
BMI	1.415	0.765–2.615	0.268
Diabetes			
No (reference)			
Yes	0.144	0.003–5.924	0.307
ASA score			
2 (reference)			
3	0.386	0.041–3.597	0.403
4	3.292	0.016–667.104	0.660
Causes			
Stones (reference)			
Stenosis	0.201	0.001–27.522	0.523
M&E classification			
1 (reference)			
2	5.449	0.245–121.257	0.284
3	32.943	0.950–1,141.760	0.053
Side			
Right (reference)			
Left	2.595	0.131–51.412	0.531
Positive urine culture			
No (reference)			
Yes	13.246	0.521–336.597	0.118
Preoperative drainage			
No (reference)			
Nephrostomy	0.366	0.018–7.391	0.512
Stenting	0.165	0.006–4.276	0.278
Surgical approach			
Open (reference)			
Laparoscopy	1.992	0.156–25.369	0.596
Surgical planning			
Elective (reference)			
Urgent	27.261	1.454–511.072	0.027

ASA = American Society of Anesthesiologists; BMI = body mass index; CI = confidence interval; M&E = monitoring and evaluation; OR = odds ratio.

*p< 0.05.

**Table 7:** Logistic regression analysis for assessing the likelihood of postoperative complications.

Variables	OR [Exp(B)]	95% CI	*p*
Age	1.062	0.958–1.177	0.255
BMI	1.012	0.707–1.448	0.948
Diabetes			
No (reference)			
Yes	0.389	0.056–2.685	0.338
ASA score			
2 (reference)			
3	0.659	0.126–3.456	0.622
4	1.883	0.028–128.598	0.769
Causes			
Stones (reference)			
Stenosis	1.135	0.132–9.747	0.908
M&E classification			
1 (reference)			
2	0.406	0.053–3.102	0.385
3	2.587	0.232–28.846	0.440
Side			
Right (reference)			
Left	0.175	0.020–1.497	0.111
Positive urine culture			
No (reference)			
Yes	1.598	0.160–15.979	0.690
Preoperative drainage			
No (reference)			
Nephrostomy	0.277	0.015–5.275	0.394
Stenting	1.752	0.274–11.211	0.554
Surgical approach			
Open (reference)			
Laparoscopy	1.578	0.314–7.921	0.579
Surgical planning			
Elective (reference)			
Urgent	2.455	0.270–22.331	0.425

ASA = American Society of Anesthesiologists; BMI = body mass index; CI = confidence interval; M&E = monitoring and evaluation; OR = odds ratio.

## 4. Discussion

We retrospectively revised our series of patients undergoing ON or LN for histopathologically confirmed XGP. Despite the fact that the minimally invasive approach is currently considered as the gold standard for both benign and malignant conditions, chronic inflammatory conditions such as XGP can make surgery extremely challenging^[18–20]^ therefore leading to the choice of an open approach. However, LN offers several advantages over ON, such as lower blood loss, shorter hospital stay, better cosmesis, and faster return to quotidian activities.

In earlier laparoscopic series, most of the authors reported excessively high conversion to open surgery and complication rates. In a small series of 9 patients with XGP, Bercowsky et al. reported that the advantages of LN did not expand to patients with XGP. They concluded that ON was faster, associated with low rates of complications, and resulted in a comparable use of analgesics, hospital stay, and recovery time.^[21]^

In recent years, laparoscopy has been revolutionized by the introduction of increasingly performing endoscopic columns and devices that warrant a magnification of the images, therefore resulting in a more precise dissection.^[22]^ In addition to technological improvements, increased surgical skills have also extended the indications of LN to include patients with XGP.^[23–26]^

Barboza et al. retrospectively examined a group of 40 patients, and demonstrated that a minimally-invasive approach was related with lower blood loss, shorter hospitalization, and lower need for intensive care admission, compared to traditional surgery.^[25]^ Similarly, in a series of 17 patients subjected to LN for XGP, Campanario-Pérez et al. underscored the feasibility of laparoscopy surgery, although it was associated with a non- negligible complication rate.^[10]^

According to our results, we observed that ON was preferentially used in those cases associated with urinary stones, while a laparoscopic approach was preferentially reserved to those cases with inveterate stenosis. This finding corroborates the hypothesis that a severe renal inflammation is frequently associated with complex inveterate urinary stones,^[27,28]^ which may lead to severe perirenal adhesions. However, it should be noted that, in multivariate analysis, the etiology of the disease did not emerge as an independent predictor of the surgical approach.

As shown by previous studies, we confirmed that patients in the LN group experienced less postoperative pain, had a faster recovery of ambulation and shorter resuming of daily activities. Interestingly, we observed no statistically significant differences in surgery time, blood loss, time to drainage removal, postoperative intensive care stay, and postoperative hospitalization between the two groups. Moreover, the postoperative bowel function restoration time was not different in the two groups.

In our experience, no conversion to open surgery occurred. Angerri et al. in a series of 96 LNs for benign pathology described 7.2% of conversion to open surgery due to the difficulty of dissecting the renal pedicle for XGP or because of major associated lesions.^[16]^

We also reported a non-negligible rate of postoperative complications *(n* = 13; 19.4%), of which, 7 (10.4%) and 6 (9%) occurred in the ON and LN groups, respectively. One death occurred in the laparoscopic group, due to septic shock. Of note, this patient suffered from decompensated diabetes. Additionally, the dense perirenal adherences substantially increased the operative time. On the first postoperative day, the patient developed severe septic shock with progressive renal failure, respiratory distress, and disseminated intravascular coagulation. The patient was promptly transferred to the critical care unit. However, despite antibiotic treatment, respiratory support, and high doses of norepinephrine, he died on the third postoperative day.

Interestingly, in his series, Campanario-Pérez et al. described two cases of septic shock.^[10]^ The first occurred during the laparoscopy and required management in the intensive care unit. The second occurred postoperatively and was secondary to a pancreatic fistula. It was unsuccessfully treated with somatostatins and empiric antibiotic therapy. Unfortunately the patient died 18 days after the procedure.

Remarkably, we detected no statistically significant difference in complication rates between the two groups, even though complications appeared slightly higher in the LN group and may achieve statistical significance in a larger population. Unfortunately, the chance of an adverse event remains a challenging issue in patients with XGP. Logistic regression analysis assessing the likelihood of complications showed that urgency exposed the patient to a higher risk of developing an intraoperative complication. However, we found no independent factors for postoperative complications. Danilovic et al. demonstrated that a higher American Society of Anesthesiology score, urgency related to a septic condition, kidney size >12 cm, and preoperative abscess were associated with a Clavien-Dindo score >1.^[29]^

This study has some limitations. First, the retrospective nature of the study may limit the significance of our findings. More specifically, the decision whether to use an open versus laparoscopic surgical approach was mainly based on the surgeon’s inclination as a result of his expertise and training rather than on preoperative-available clinical and radiological findings. In addition, our institution is a tertiary center with elevated laparoscopic expertise. This may have influenced the outcomes of our series and may have reduced the generalizability of our results to small centers.

To the best of our knowledge, this is the largest case series reporting the outcomes of ON and LN for XGP that has been published. We presented a realistically large and evenly balanced number of subjects in both ON and LN groups. Our retrospective analysis confirms the results of previously published contemporary experiences showing the feasibility of a laparoscopic approach in experienced hands.^[30]^ Despite the case of septic shock, all other complications were successfully managed, suggesting that LN has an acceptable morbidity.

Based on our results, LN may result in less postoperative pain, faster recovery of ambulation, and shorter resuming of daily activities relative to ON. Therefore, LN should be considered as a preliminary approach for XGP at least in laparoscopically experienced centers. However, a careful evaluation of preoperative CT findings should always be performed in order to define the risk-benefit ratio associated with both surgical strategies. Ideally, randomized controlled trials are desirable to confirm the present results.

## Acknowledgments

None.

## Statement of ethics

The protocol of this research was accepted by the institute’s committee on human research. All the subjects consented to and signed informed consent. All procedures involving human subjects were performed according to the ethical standards of the institutional investigation committee and with the 1964 Helsinki declaration and its later amendments.

## Conflict of interest statement

The authors report no conflicts of interest.

## Funding source

None.

## Author contributions

FC: Protocol creation and manuscript writing;

FP: Manuscript editing and interpretation of data;

MF: Data analysis;

CM: Data collection and managing;

RG: Data collection and management;

MF: Data analysis and manuscript writing;

GL: Critical revision of the paper and statistical analysis check;

PF: Project progress.
